# Mid-way and post-intervention effects on potential determinants of physical activity and sedentary behavior, results of the HEIA study - a multi-component school-based randomized trial

**DOI:** 10.1186/1479-5868-9-63

**Published:** 2012-05-29

**Authors:** Ingunn H Bergh, Mona Bjelland, May Grydeland, Nanna Lien, Lene F Andersen, Knut-Inge Klepp, Sigmund A Anderssen, Yngvar Ommundsen

**Affiliations:** 1 Department of Coaching and Psychology, Norwegian School of Sport Sciences, Oslo, Norway; 2 Department of Sports Medicine, Norwegian School of Sport Sciences, Oslo, Norway; 3 Department of Nutrition, Faculty of Medicine, University of Oslo, Oslo, Norway

**Keywords:** Moderation, Adolescents, Obesity prevention, Intervention, Social-ecological model, Effect, Randomized controlled trial

## Abstract

**Background:**

There is limited knowledge as to whether obesity prevention interventions are able to produce change in the determinants hypothesized to precede change in energy balance-related behaviors in young people. The aim of this study was to evaluate the effect of a multi-component intervention on a wide range of theoretically informed determinants of physical activity (PA) and sedentary behavior (SB). Moderation effects of gender, weight status and parental education level and whether the perceived intervention dose received influenced the effects were also explored.

**Methods:**

The HEIA study was a 20-month school-based, randomized controlled trial to promote healthy weight development. In total, 1418 11-year-olds participated at baseline and post-intervention assessment. Enjoyment, self-efficacy, perceived social support from parents, teachers and friends related to PA, perceived parental regulation of TV-viewing and computer/game-use and perceived social inclusion at schools were examined by covariance analyses to assess overall effects and moderation by gender, weight status and parental education, mid-way and post-intervention. Covariance analyses were also used to examine the role of intervention dose received on change in the determinants.

**Results:**

At mid-way enjoyment (p = .03), perceived social support from teachers (p = .003) and self-efficacy (p = .05) were higher in the intervention group. Weight status moderated the effect on self-efficacy, with a positive effect observed among the normal weight only. At post-intervention results were sustained for social support from teachers (p = .001), while a negative effect was found for self-efficacy (p = .02). Weight status moderated the effect on enjoyment, with reduced enjoyment observed among the overweight. Moderation effects for parental education level were detected for perceived social support from parents and teachers. Finally, positive effects on several determinants were observed among those receiving a high as opposed to a low intervention dose.

**Conclusion:**

The intervention affected both psychological and social-environmental determinants. Results indicate that social support from teachers might be a potential mediator of PA change, and that overweight adolescents might be in need of specially targeted interventions to avoid reducing their enjoyment of PA. Further studies should continue to assess how intervention effectiveness is influenced by the participants’ self-reported dose of intervention received.

## Background

Engagement in physical activity (PA) and sedentary behavior (SB) are regarded as two important factors in obesity prevention programs [1]. School-based interventions to increase PA and reduce SB may promote a healthy weight development, but the results are inconsistent [2]. One possible explanation for low efficacy or effectiveness is limited evidence as to how interventions induce behavior change [3] and for whom interventions are effective [4]. More research in order to improve the understanding of PA and SB change is called for [5,6].

According to the mediating framework, change in hypothesized determinants is a prerequisite for change in behavior [7]. Hence, potential determinants could be considered endpoints in themselves and thus would seem important to identify [8-10]. Knowledge about change in determinants will help tease out important influences that may pave the way for behavior change at a later stage, and by identifying changes in potential determinants one may avoid running the risk of underestimating important intervention effects in cases when behavior change cannot be observed.

Only a limited number of PA interventions targeting adolescents have reported the effects of change in determinants, and in about half of the identified studies no effect on these were detected [11]. However, changes in self-efficacy and enjoyment of PA have been identified, and these determinants have also been proven to mediate PA change in children and adolescents [5,6]. Effect on change in inter-personal determinants like social support for PA is less investigated [5,6,11], and no one seems to have examined change in potential inter-personal determinants of SB such as perceived parental regulation of TV-viewing or computer/game-use [5] or social inclusion related to the school context [12]*.*

It is argued that testing effect modifiers should become common practice in intervention studies [13,14]. In school based interventions targeting energy balance-related behaviors, gender seems to be the most convincing moderator while findings for potential effect modifiers such as weight status and socio-economic status (SES) are inconsistent [15]*.* Examining moderating influences on change in potential determinants of behavior change will help identify for whom an intervention is effective or not. Consequently, it will provide knowledge about the need to target subgroups differently when designing and implementing intervention [3,16]. Moreover, how much of the intervention is received by the participants might also influence the effect of an intervention [17]. Hence, examination of exposure and participation has been called for [18,19]*.*

The HEalth In Adolescents (HEIA) study was a 20-month intervention designed to promote healthy weight development among adolescents (11-13-year-olds) through change in PA, SB and dietary behaviors. Change in the behaviors was targeted through multilevel intervention strategies hypothesized to influence a wide range of psychological and social-environmental determinants [20]. The selection of potential determinants of PA and SB change was based on a social-ecological approach including determinants at the personal/psychological, social and environmental level as embedded in the conceptual model of the HEIA study [20,21]*.* Previously in the HEIA study weight status has been found to moderate the association between correlates and PA [22] and to moderate change in SB among boys, while no moderation effect of parental education was detected [23]*.*

Hence, the aims of this paper were threefold; 1) to examine the effect of the HEIA intervention upon theoretically informed psychological and social-environmental determinants of PA and SB change measured mid-way (after 8 months) and post-intervention (after 20 months), 2) to investigate moderating effect of gender, weight status and SES on the set of determinants and 3) to explore whether the degree of intervention exposure and participation influenced these outcomes.

## Methods

### Study design and population

**Figure 1:**
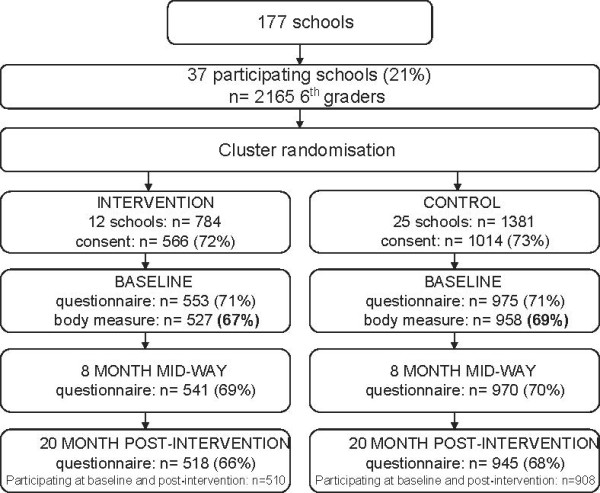
Flow diagram of recruitment, randomization, consent received and participation of adolescents in the HEIA study.

Schools were recruited from towns/municipalities in seven counties in the south-eastern part of Norway. For logistic reasons schools had to have at least 40 pupils enrolled in 6^th^ grade which qualified 177 schools to receive an invitation. Thirty-seven schools accepted the invitation, and all the 6^th^ graders (n = 2165) in the attending schools and their parents/legal guardians were invited to participate (Figure 1) [20]. Of these, 1580 (73%) returned a parental signed informed consent form.

A cluster randomized design was used to evaluate the intervention; 12 schools were randomly assigned by simple drawing to the intervention group and 25 to the control group. The baseline data collection was conducted in September 2007 (in the beginning of 6^th^ grade), the mid-way assessment was conducted in May 2008 (at the end of 6^th^ grade; 8 months past baseline). All three assessments were administered over approximately four weeks, with parallel assessments in the intervention and control group.

The adolescents who participated in both the baseline and post-intervention data collections are included in the analyses; in total 1418 (908 in Control; 510 in Intervention; 89% of the 1580 returning the consent forms), and of those 1384 participated in the mid-way assessment (885 in Control; 499 in intervention; 87% of the1580 returning the consent forms). Comparisons of the outcomes and demographic variables between those participating only at baseline and those participating at a) baseline and post-intervention and b) all three assessments, revealed significant lower values for perceived social inclusion at school (p = .02) and a higher proportion of overweight adolescents (p = .007 and p = .01) among those lost to follow-up between baseline and post-intervention (n = 110) and between baseline and both mid-way and post-intervention (n = 144) (data not shown).

### Intervention

A detailed description of the study design, the development and all the parts of the intervention (dietary, PA and SB) have been presented elsewhere [20]*.* The intervention consisted of a mix of individual-, group- and environmental strategies and components. All the PA and SB components and the targeted determinants are listed in additional file 1: Table S1 xand it illustrates how several of the components were directed towards change in multiple determinants. The first part (in 6^th^ grade) emphasized activities that were supposed to make PA enjoyable and create a sense of efficacy for PA. Most of the PA components included interactions with class-mates to facilitate social cohesion and support. In the second part of the intervention (in 7^th^ grade) some components targeting SB were included.

In short, the 6 ^th^ grade components were: 1) One theoretical class-room lesson over 90 minutes, concerning PA and dietary behaviors in relation to the energy balance equation (the other four lessons focused primarily on dietary behavior). 2) Short PA breaks during lessons (once a week), 3) two active-commuting-to-school campaigns, 4) an “activity box” with sport- and play equipment for use during recess (including e.g. frisbees, jump-ropes, elastic bands, hockey-sticks, several types of balls). 5) fact sheets for parents (3 specifically on PA, 6 in total), and 6) one inspirational course for teachers responsible for the physical education (PE) classes in which the teachers practiced new ideas for lessons that they were to try out in the PE-classes. The lessons focused on type of novel, enjoyable games and activities with the intention to keep all the students in moderate to high intensity most of the class-time, and was based on the SPARK program [24]. The 7^th^ grade components included: 1) an extensionx of PA breaks and 2) the two active-commuting to school campaigns in which the adolescents were provided with pedometers. The focus in this “pedometer challenges” was to stimulate both active commuting to schools as well as more daily PA. 3) Some of the equipment in the “activity box” was replaced due to loss from wear and tear. 4) A second inspirational course for PE-teachers was organized providing the teachers with additional lessons to try out in PE-classes (some included use of pedometers). 5) A computer tailoring program targeting SB, PA and dietary behaviors was added to the intervention with four sessions in total, including one on SB (both TV-viewing and computer/electronic game-use) and one on PA. 6) In addition new parental fact sheets targeting both PA and SB (3 on PA, 1 on TV and computer/game-use, 9 in total) were distributed to the parents now including child–parent homework assignments.

Each school year the intervention was initiated by a kick-off meeting with the involved teachers. The purpose of these meetings was to ensure that the whole team of teachers knew the rationale, was familiar with the various intervention elements and were motivated to implement the components and support the targeted behaviors. During the school year the participating teachers received external support in form of short monthly e-mail reminders from the HEIA study group. All the adolescents in the intervention schools took part in the intervention, but only those with consent took part in the data collection. The control schools followed the regular Norwegian school curriculum including PE-classes (2 X 45 min/week), but they were not restricted with respect to developing their own PA, SB or dietary initatives**.**

Ethical approval and research clearance was obtained from the Regional Committees for Medical Research Ethics and the Norwegian Social Science Data Service.

### Measurements

#### Questionnaire data

The adolescents self-reported the potential determinants of PA and SB, and gender in an internet-based questionnaire which took about 45 minutes to complete. Process evaluation questions tapping into the adolescents’ perception of exposure and participation in the intervention were included in the questionnaire for those in the intervention group at both the mid-way and post-intervention assessments. The questionnaires were completed in schools with trained personnel present.

#### Outcome measures

The outcome measures included nine hypothesized psychological and social-environmental determinants of PA and SB change. The psychological variables included an abbreviated and slightly modified version of the *Enjoyment ofPAscale*[25] and the *self-efficacy related to barriers forPA* scale based on previous studies [26,27]. These changes were induced to keep the questionnaire at a reasonable length and to obtain satisfactory reliability estimates. The social-environmental variables were: *Perceived social supportfor PAfrom parents* assessed by five items; *perceived social supportfor PAfrom friends* assessed by three items [28]; *Perceived social supportfor PAfrom teacher* taken from a pilot study within the European Youth Heart Study [29] assessed by three items; *Perceived Environmental opportunities to be physically active* from Sallis et al. [28] with one added item and assessed by 4 items; *Perceived parental regulation of TV-viewing* and *perceived parental regulation of computer/game-use*, each assessed by four items modified from Hardy et al. 2006 [30] and *Perceived social inclusion related to the school and class environment* assessed by six items based on a “social capital measure” (related to people in my area/neighborhood) developed by Hume et al. 2009 [12] modified to capture the quality of relationship with peers at school both within and outside the classroom (degree of closeness and willingness to ask for/provide help when necessary). All the items were rated on a 5-point Likert-type scale coded 1 (lowest) to 5 (highest), and phrased “totally disagree” to “totally agree” with a neutral midpoint, except for the social support constructs which were phrased “almost never or never”, “1 to 2 times a week”, “3 to 4 times a week”, “almost every day” and “every day”.

All the nine outcomes were assessed at baseline and post-intervention, while enjoyment, self-efficacy, perceived social support from parents and teachers were measured also at the mid-way assessment. All variables obtained acceptable internal reliability values (Cronbach’s alpha) at baseline (range 0.62-0.82), mid-way (range 0.68-0.75) and post-intervention (range 0.67-0.85). Examples of the items, the procedure for computing the composite scores, the results from a separate test-retest study showing moderate to high test-retest values (ICC), and the theoretical models from which these variables have been derived, have been reported elsewhere [22]*.*

#### Weight status and parental education

Height and weight of the adolescents were measured objectively, and the baseline values were used to categorize the adolescents as normal weight and overweight by the age and gender specific body mass index cut-offs values proposed by the International Obesity Task Force [31]. Due to the low proportion of obese in the sample at baseline (1.5% in total sample; 1.6% in Control; 1.3% Intervention), the overweight and obese were treated as one group in the analyses, and are referred to as the “overweight group” throughout the paper. Details of the procedures and test-retest values of the anthropometric measurements have been reported elsewhere [20,32]*.* Parental education was used as an indicator of SES, and was reported by the parents on the informed consent form. Parental education level was categorized into three levels: 12 years or less, between 13 and 16 years and 16 years or more, and the parents with the highest education was used or else the one available.

#### Perceived intervention dose received

To obtain information about the intervention dose received [17], the adolescents answered process questions about degree of exposure to or participation in the PA and SB intervention components both mid-way (6 questions) and post-intervention (7 questions). The response categories were yes (1) or no (0), while three of the questions had three or more response categories and were dichotomized into yes/no (Table 1). All the intervention components were formatted as a package and supposed to be implemented as such with components expected to mutually reinforce each other. Therefore, a sumscore for the total dose received at mid-way and post-intervention were calculated by adding and averaging the numbers of questions, giving scores ranging from 0.00 to 1.00 (0 = minimum and 1.00 = maximum degree of exposure/participation). By inspecting the distribution of the scores, we set a score equal or above 0.75 (75%) to represent a “high” dose received of the intervention and a score lower than 0.75 to represent a “low” dose received. The questions, the coding and the distribution (%) for the specific process questions and the perceived total intervention dose received mid-way and post-intervention are presented in Table 1.

**Table 1:** Exposure to/participation in the PA and SB intervention components and total perceived intervention dose received

	**Response categories**	**Coded: no (0)/yes (1)**	**Yes % (n)**
**Mid-way: questions assessing perceived exposure/participation**			
Have you completed in class assignment about diet, PA and SB?	Yes/no		92.9 (446)
Have you noticed HEIA-posters in the classroom?	Yes/no		82.2 (402)
Have you participated in the active transport campaign?	Yes/no		89.0 (435)
Have you participated in one or more HEIA-breaks with PA?	Yes/no		82.7 (401)
Have you used the equipment in the “activity box” during recess?	Each school day or almost each school day **(yes)**/once a week, rarely or never **(no)**		41.7 (287)
Have you used movement bands in the PE-classes?	Yes/no or does not know (**no**)		47.5 (235)
***Total intervention dose received midway (n = 492)***	***Range:***	***0.00-1.00***	***High dose ≥0.75***
			55.5% (273)
**Post-intervention:**			
Have you completed the computer tailoring session about PA?	Yes/no		88.1 (436)
Have you completed the computer tailoring session about TV and computer/game-use?	Yes/no		87.2 (430)
Have you noticed HEIA-posters in the classroom?	Yes/no		75.2 (377)
Have you participated in a “pedometer challenge” (related to active commuting/active daily living)?	yes twice (**yes**)/just once or no (**no**)		83.4 (418)
Have you participated in one or more HEIA-breaks with PA during school lessons?	Yes/no		78.4 (389)
Have you used the equipment in the “activity box” during recess?	Each school day or almost each school day **(yes)**/once a week, rarely or never **(no)**		39.2 (197)
Have you used “Basse” (ball made of rubber bike wheels) during school hours?	Yes everyday **(yes)**/one day pr week/no or does not know what Basse is **(no)**		26.8 (134)
***Total intervention dose received post intervention (n = 503)***	***Range:***	***0.00-1.00***	***High dose ≥0.75***
			31.0% (156)

PA = Physical activity, SB = Sedentary behavior.

Sample: Norwegian adolescents 11-13 year-olds from the HEIA study.

#### Data analyses

Chi-square and independent t-tests were used to analyze drop-outs and to compare baseline characteristics and outcomes between groups (control vs. intervention, and low vs. high intervention dose received).

Clustering effects due to schools being the unit of recruitment was checked by the linear mixed model procedure. Only 0-4% of the unexplained variance in the outcomes was on the group (i.e. school) level, except for perceived social support from teachers being slightly higher (9%). Hence, for perceived social support from teachers we did check results by the linear mixed model procedure, adjusting for the school effect. Given that the same pattern of results was revealed when taking into account the potential clustering effect of school, it was decided to run and present all further analyses without adjusting for the clustering effect.

In the main analyses, the overall effects from baseline to mid-way and from baseline to post-intervention were investigated in two steps by one-way ANCOVA, with the mid-way and post-interventions values for the outcome measures as dependent variables, baseline values as covariates and group (intervention vs. control) as the independent variable.

Next, in separate analyses moderating influences on the effects were examined by two-way ANCOVA both at mid-way and at post-intervention for the following variables: gender, weight status (normal weight vs. overweight) and parental education level (≤12 years, 13–16 years, >16 years). For cases in which significant moderating influences were revealed, subgroup analyses were carried out using one way ANCOVA to test for differences between the control and intervention for each subgroup.

Lastly, the effect of the perceived intervention dose received (low vs. high) on the determinants were analyzed first at mid-way and then at post-intervention, with one-way ANCOVA within the intervention schools only. The mid-way and post-interventions values for the outcome measures were entered as dependent variables, baseline values as covariates and intervention dose received (high vs. low) as the independent variable.

Data were checked to ensure there were no violations of the assumptions for the ANCOVA analyses. All statistical analyses were performed by IBM SPSS Statistics, version 18.0 (IBM Corp., Somers, New York, USA). The significance level was set at p < .05 for all analyses, except for the interaction tests where p < .10 was used.

## Results

Table 2 shows the baseline characteristic for the study population by condition. No significant differences between the intervention and control group were revealed for the demographic variables.

**Table 2:** Baseline demographics and weight status for the control and intervention group

	**Control**	**Intervention**	
	(n^†^ = 908)	(n^†^ = 510)	p
**Age** (mean; SD)	11.2 (0.27)	11.2 (0.26)	.38
**Gender**			
Girls (%)	47.8	49.6	.54
Boys (%)	52.2	50.4	
**Weight status**			
Normal weight (%)	85.5	88.6	.12
Overweight (%)	14.5	11.4	
**Parental education**			
<12 years (%)	31.1	26.2	.15
13-16 years (%)	35.8	37.7	
>16 years (%)	33.1	36.1	

p = Independent *t*-test (age) and chi-square.

^†^n varies slightly for weight status and parental education.

Sample: Norwegian adolescents 11-13 year-olds from the HEIA study.

In Table 3 the means (SD) for all the outcomes are shown at the baseline, mid-way and post-intervention assessments. There were no significant differences between the intervention and control group at baseline.

**Table 3:** Baseline, mid-way and post-intervention characteristics for PA and SB determinants in the control and intervention group

	**Baseline**					**Mid-way**				**Post-intervention**			
	Control (n^†^ = 908)		Intervention (n^†^ = 510)			Control (n^†^ = 885)		Intervention (n^†^ = 499)		Control (n^†^ = 908)		Intervention (n^†^ = 510)	
	Mean	SD	Mean	SD	p	Mean	SD	Mean	SD	Mean	SD	Mean	SD
Enjoyment	4.13	(0.76)	4.09	(0.77)	0.35	4.08	(0.75)	4.13	(0.78)	3.96	(0.83)	3.88	(0.90)
Self-efficacy	3.86	(0.76)	3.86	(0.79)	0.80	3.93	(0.75)	3.99	(0.78)	3.86	(0.82)	3.77	(0.89)
Social support from parents	2.37	(0.76)	2.38	(0.76)	0.83	2.45	(0.81)	2.42	(0.77)	2.31	(0.81)	2.24	(0.78)
Social support from teachers	1.68	(0.70)	1.76	(0.83)	0.07	1.61	(0.65)	1.75	(0.77)	1.47	(0.62)	1.61	(0.73)
Social support from friends	2.96	(1.00)	2.96	(1.04)	0.95					2.80	(0.99)	2.82	(0.97)
Environmental opportunities for PA	4.26	(0.77)	4.27	(0.76)	0.79					4.11	(0.89)	4.20	(0.90)
Parental regulation TV-viewing	3.64	(0.96)	3.68	(0.93)	0.43					3.41	(1.07)	3.38	(1.05)
Parental regulation computer/game-use	3.55	(0.99)	3.53	(1.01)	0.78					3.28	(1.11)	3.28	(1.10)
Social inclusion at school	4.43	(0.61)	4.39	(0.62)	0.15					4.36	(0.69)	4.32	0.77

PA = Physical activity, SB = Sedentary behavior, SD = Standard deviation.

^†^n varies slightly for the different variables.

p-values for independent *t*-test between control and intervention group at baseline.

Range 1–5 (lowest to highest with a neutral midpoint) for all variables.

Sample: Norwegian adolescents 11-13 year-olds from the HEIA study.

### Main effects

Table 4 shows the effect of the intervention on the four determinants assessed mid-way, and the effect on all the nine determinants measured post-intervention.

**Table 4:** Effects on determinants for PA and SB mid-way and post intervention

	**Control**		**Intervention**		
	Mean^†^	95% CI	Mean^†^	95% CI	p
**Mid-way**					
Enjoyment	4.07	(4.03, 4.12)	4.15	(4.10, 4.21)	**.03**
Self-efficacy	3.94	(3.90, 3.98)	4.01	(3.95, 4.06)	**.05**
Perceived social support from parents	2.44	(2.40, 2.49)	2.43	(2.37, 2.49)	.77
Perceived social support from teachers	1.63	(1.58, 1.67)	1.73	(1.67, 1.78)	**.003**
**Post-intervention**					
Enjoyment	3.95	(3.90, 4.00)	3.89	(3.82, 3.96)	.19
Self-efficacy	3.86	(3.81, 3.91)	3.76	(3.70, 3.83)	**.02**
Social support from parents	2.31	(2.26, 2.36)	2.24	(2.18, 2.31)	.10
Social support from teachers	1.48	(1.44, 1.52)	1.59	(1.54, 1.65)	**.001**
Social support from friends	2.80	(2.74, 2.86)	2.82	(2.74, 2.90)	.76
Environmental opportunities for PA	4.12	(4.07, 4.18)	4.21	(4.13, 4.28)	.08
Parental regulation TV-viewing	3.42	(3.35, 3.48)	3.37	(3.29, 3.46)	.40
Parental regulation computer/game-use	3.28	(3.21, 3.34)	3.30	(3.21, 3.39)	.68
Social inclusion at school	4.36	(4.31, 4.40)	4.33	(4.27, 4.39)	.47

PA = Physical activity, SB = Sedentary behavior, CI = Confidence intervals.

One-way Ancova analyses.

^**†**^ Adjusted for baseline values of determinants.

Sample: Norwegian adolescents 11-13 year-olds from the HEIA study.

At mid-way there were small significant positive effects of the intervention on both enjoyment and perceived social support from teachers, and a borderline significant positive effect for self-efficacy. No effect on perceived social support from parents was observed (Table 4). For enjoyment the change was expressed as a small increase in the intervention group, and a small decrease in the control group (Table 3). The level of social support from teachers stayed about the same in the intervention group, but there was slight decrease in the control group (Table 3). For self-efficacy the borderline significant change reflected a slightly greater increase in the intervention group compared to the control group.

At post intervention a negative intervention effect was detected for self-efficacy (Table 4), reflecting a small reduction in self-efficacy in the intervention group with no change in the control group (Table 3). A positive intervention effect was revealed for perceived social support from teachers (Table 4) seen as a somewhat smaller reduction in the intervention group compared to the control (Table 3**)**. No effects on any of the other determinants were observed.

### Interaction and subgroups effects

**Figure 2:**
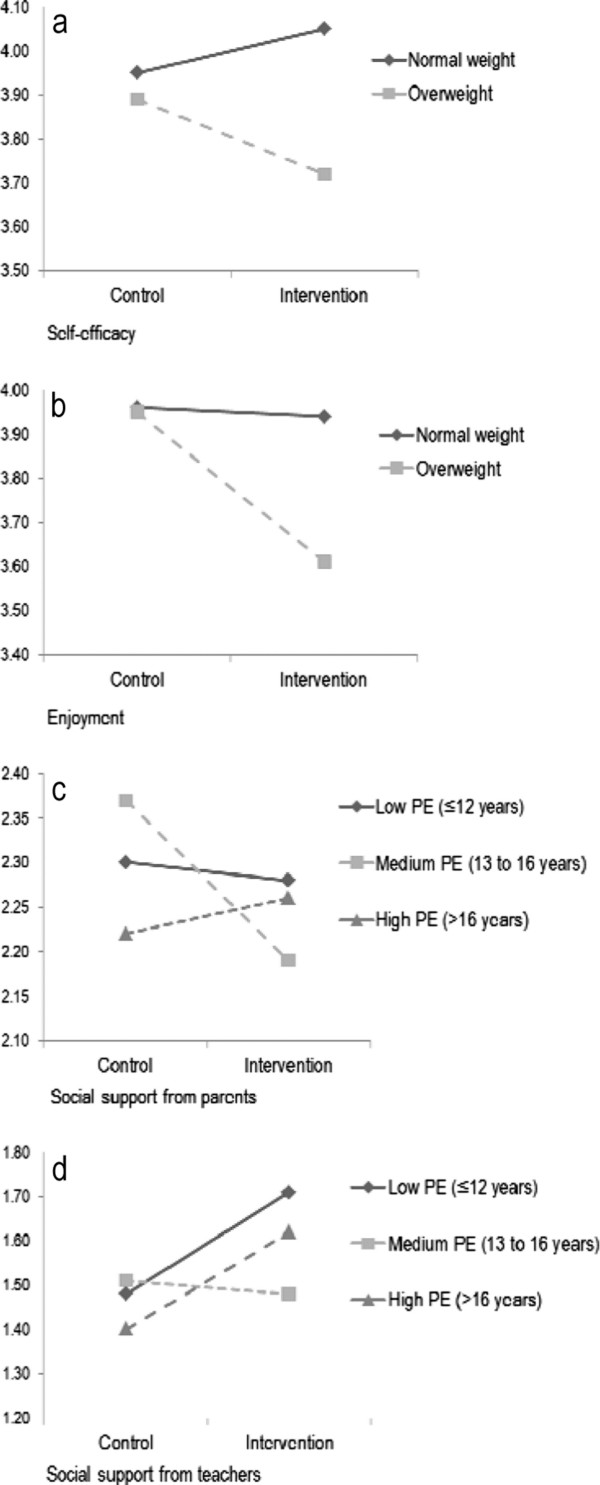
**(a) Interaction effect of weight status on change in self-efficacy mid-way.(b)** Interaction effect of weight status on change in enjoyment post-intervention. **(c)** Interaction effect of parental education level on change in social support from parents post-intervention. **(d)** Interaction effect of parental education level on change in social support from teachers post-intervention.

At mid-way no interaction effects of gender or parental education were found on change in the four determinants assessed. However, weight status moderated the effect of the intervention on change in self-efficacy (p = 0.01) (Figure 2a). Similarly, no interaction effects of gender was found at post-intervention, but weight status moderated the effect on change in enjoyment (p = .02) (Figure 2b). Also, parental education level moderated the effect on pre to post-intervention change in perceived social support from parents (p = .07) (Figure 2c) and from teachers (p = .003) (Figure 2d).

The results of the corresponding subgroup analyses mid-way and post-intervention are shown in Table 5**.** At mid-way the effect of the intervention on self-efficacy was different for the normal weight and overweight adolescents. While the normal weight reported higher self-efficacy, a trend (non-significant) for reporting lower self-efficacy among the overweight was observed. Post-intervention, the effect on enjoyment differed for the normal weight and overweight. While no effect on change in enjoyment among the normal weight was observed, the overweight group reported a reduction in enjoyment.

**Table 5:** Effects on determinants for PA and SB, by weight status and parental education level

		**Control**	**Intervention**		**Control**		**Intervention**		
		Baseline Crude Mean SD	Baseline Crude Mean SD	p^1^	Adjusted Mean^**†**^	95% CI	Adjusted Mean	95% CI	p^2^
**Mid-way**									
Self-efficacy	Normal weight	3.89 (0.74)	3.87(0.78)	.63	3.96	(3.92, 4.01)	4.06	(4.00, 4.12)	**.01**
	Overweight	3.74 (0.85)	3.69 (0.82)	.70	3.80	(3.70, 3.91)	3.63	(3.46, 3.80)	.09
**Post-intervention**									
Enjoyment	Normal weight	4.14 (0.75)	4.10 (0.77)	.45	3.96	(3.90, 4.02)	3.94	(3.86, 4.02)	.67
	Overweight	4.08 (0.84)	3.99 (0.71)	.49	3.92	(3.78, 4.06)	3.58	(3.37, 3.79)	**.009**
Social support from parents	Low PE (≤12 years)	2.32 (0.80)	2.50 (0.93)	.08	2.30	(2.21, 2.40)	2.28	(2.15, 2.42)	.81
	Medium PE (13 to 16 years)	2.44 (0.80)	2.35 (0.67)	.20	2.38	(2.30, 2.46)	2.21	(2.11, 2.32)	**.01**
	High PE (>16 years)	2.35 (0.69)	2.31 (0.68)	.52	2.20	(2.13, 2.28)	2.24	(2.15, 2.34)	.53
Social support from teachers	Low PE (≤ 12 years)	1.76 (0.76)	1.72 (0.91)	.70	1.49	(1.41, 1.57)	1.72	(1.60, 1.83)	**.001**
	Medium PE (13 to 16 years)	1.73 (0.73)	1.66 (0.70)	.28	1.51	(1.44, 1.58)	1.49	(1.40, 1.58)	.66
	High PE (>16 years)	1.57 (0.61)	1.87 (0.86)	**<.001**	1.40	(1.33, 1.47)	1.60	(1.51, 1.69)	**.001**

PA = Physical activity, SB = Sedentary behavior, SD = Standard deviation, CI = Confidence interval, PE = parental education level.

One-way Ancova analyses,

n for Control vs. Intervention in analyses for subgroups: normal weight (737–752 vs. 404–417), overweight (121–125 vs. 51–53), low PE (261–266 vs 120), medium PE (302–311 vs 178), high PE (285–287 vs.171).

^**†**^ Adjusted for baseline values.

p^1^ = Independent *t*-test, p^2^ = One way Ancova.

Sample: Norwegian adolescents 11-13 year-olds from the HEIA study.

In addition, at post-intervention the effect on perceived social support from parents and from teachers differed by the adolescents’ SES. Adolescents with medium parental education reported lower social support from parents compared to the control group. There was no intervention effect on change in perceived social support from parents among those with the lowest and highest parental education. In contrast, there was a positive intervention effect on change in perceived social support from teachers among those with lowest and highest parental education. No intervention effect on change in perceived social support from teachers was found among those with medium parental education.

### Perceived intervention dose received

At mid-way 273 (55.5%) of the adolescents in the intervention group reported a high intervention dose received, whereas 156 (31.0%) reported this at the time of the post-intervention (Table 1). Table 6 show the influence on change in the examined determinants within the intervention group.

**Table 6:** Influence of intervention dose received on the determinant for PA and SB

	**Intervention dose received**	**Crude Mean (SD) Baseline**	**p**^**1**^	**Adjusted Mean**	**95% CI**	**p**^**2**^
**Mid-way** (low n = 219, high n = 273)*						
Enjoyment	Low	4.03 (0.82)	.12	4.03	(3.94, 4.12)	**.001**
	High	4.13 (0.72)		4.22	(4.15, 4.30)	
Self-efficacy	Low	3.72 (0.85)	**.001**	3.91	(3.83, 4.00)	**.005**
	High	3.97 (0.72)		4.07	(4.00, 4.15)	
Perceived social support from parents	Low	2.30 (0.75)	.65	2.39	(2.30, 2.48)	.27
	High	2.43 (0.77)		2.46	(2.38, 2.54)	
Perceived social support from teachers	Low	1.66 (0.71)	**.02**	1.62	(1.53, 1.71)	**<.001**
	High	1.82 (0.89)		1.86	(1.78, 1.94)	
**Post-intervention** (low n = 347, high n = 156)*						
Enjoyment	Low	4.03 (0.79)	**.01**	3.82	(3.74, 3.91)	**.01**
	High	4.22 (0.67)		4.02	(3.89, 4.15)	
Self-efficacy	Low	3.79 (0.82)	.001	3.72	(3.63, 3.80)	.07
	High	4.01 (0.66)		3.86	(3.73, 3.99)	
Social support from parents	Low	2.33 (0.77)	.05	2.21	(2.13, 2.29)	.06
	High	2.48 (0.74)		2.34	(2.23, 2.46)	.
Social support from teachers	Low	1.72 (0.80)	.09	1.57	(1.49, 1.64)	.07
	High	1.86 (0.89)		1.69	(1.58, 1.80)	
Social support from friends	Low	2.92 (1.05)	.18	2.75	(2.66, 2.85)	**.02**
	High	3.05 (0.98)		2.96	(2.82, 3.11)	
Perceived environmental opportunities	Low	4.20 (0.76)	**.002**	4.15	(4.06, 4.24)	**.02**
	High	4.43 (0.71)		4.35	(4.22, 4.48)	
Parental regulation TV-viewing	Low	3.68 (0.92)	.88	3.33	(3.23, 3.43)	.07
	High	3.69 (0.92)		3.50	(3.35, 3.66)	
Parental regulation computer/game-use	Low	3.51 (0.99)	.5	3.27	(3.15, 3.38)	.41
	High	3.58 (1.04)		3.35	(3.19, 3.52)	
Social inclusion at school	Low	4.35 (0.63)	.08	4.26	(4.18, 4.34)	**.009**
	High	4.46 (0.56)		4.45	(4.33, 4.57)	

PA = Physical activity, SB = Sedentary behavior, SD = Standard deviation, CI = Confidence interval.

Only participants within the intervention schools included in the analyses.

*n varies slightly for the different outcomes due to the incomplete data.

^**†**^ Adjusted for baseline values.

p^1^ = Independent *t*-test, p^2^ = One-way Ancova.

Sample: Norwegian adolescents 11-13 year-olds from the HEIA study.

Compared to adolescents reporting a low intervention dose received those reporting a high dose showed significantly higher adjusted mean values on enjoyment, self-efficacy and perceived social support from teachers. Parallel dose specific post-intervention findings were observed for enjoyment, perceived social support from friends, perceived environmental opportunities for PA and for perceived social inclusion at school (Table 6).

## Discussion

This study examined mid-way and post-intervention effects of a 20 month school-based obesity prevention intervention upon a wide range of determinants of PA and SB. In addition moderating effects of gender, weight status and parental education were assessed and the influence of perceived intervention dose received by the participants on the determinants was explored.

For the whole sample favorable effects on both psychological and social-environmental determinants of PA were found mid-way. However, post-intervention the effect was only sustained for social support from teachers, whereas an unexpected negative effect on self-efficacy for PA was revealed. The intervention did not affect any of the SB determinants. Moderation effects of weight status and parental education were observed, and subgroup analyses showed that the intervention did not work equally well in all subgroups. In addition, analyses of intervention dose received indicated that the effect on the determinants was influenced by the adolescents’ reported degree of exposure to and participation in the intervention.

### Psychological determinants

Most of the intervention components that targeted the adolescents emphasized promoting enjoyment of PA, possibly explaining the overall positive mid-way effect on enjoyment. Our finding are consistent with the results of a 12 week long intervention among younger girls [33] and would seem encouraging given that enjoyment of PA has been shown to be of great importance for activity initiation and continued interest [34]. Moreover, enjoyment has been identified as a mediator of PA change in adolescent girls [35]. However, in accordance with results from a longer lasting intervention with a similar age group as the current one [36] no overall favorable effect on enjoyment was seen post-intervention, while a clear, reduction in enjoyment was detected among the overweight. The latter result would seem troublesome, and might reflect that various intervention activities have not met with the needs of those being overweight. *.*

Even though there was an overall marginally positive mid-way effect for self-efficacy, the subgroup analyses revealed a positive effect among the normal weight only, while there was a tendency towards a negative effect on self-efficacy among the overweight. Despite focusing on low threshold intervention activities, the negative mid-way trend for self-efficacy together with the reduction of enjoyment seen post-intervention among the overweight could well reflect that the overweight group has not felt at ease with these activities provided over time. Indeed, a sense of competence and feeling efficacious has been shown to be a key factor for enjoying PA [37,38]**.** Alternatively, social comparison processes with those being normal weight might have led to unfavorable self-perceptions and enjoyment among the overweight [39].

As to self-efficacy post-intervention, the results showed an effect in the undesired direction for the whole sample. However, this type of unexpected result has also been seen in other studies [40,41]. Due to the comprehensive nature of our intervention we cannot draw conclusion to which intervention components the effect can be attributed. However, it could well be that participants were more unaware of barriers to PA change in the first school-year of the intervention, but as the intervention moved along they might have become more aware of and realistic about barriers for PA.

### Social-environmental determinants

While no overall effects on social support from friends or parents were seen mid-way or post-intervention, the negative post-intervention change for perceived social support from parents among the adolescent with medium level of parental education is not readily explainable. While one could assume that this result was due to baseline differences between control and intervention group this was, however, not the case (Table 5).

To our knowledge, this is the first study to report a positive effect on perceived social support from teachers and this was observed both mid-way and post-intervention. These results are encouraging because teachers are in the position to reach most adolescents and hold the role as change facilitators in most school-based interventions. The post-intervention subgroup differences for parental education level revealed that the effect on social support from teachers was predominantly seen among adolescents with lower and higher parental education background. Most importantly, these results indicate that teachers also seem to be able to reach children with lower socio-economic status when it comes to providing support for PA, and that teacher support may be a source of social influence that holds the potential to influence the social gradient that seems to exist concerning PA among adolescents [42]*.* The yearly kick-off meetings for the teachers targeting the whole teacher team at each school to support the intervention might have contributed to a sense of enhanced support from teachers. At the same time low baseline values means that there was greater room for improvement in social support from teachers compared to many of the other determinants.

The intervention did not have an impact on determinants for SB post-intervention (perceived regulation of parental TV-viewing and computer/game-use), even though mid-way effects on TV-viewing and computer/game-use among girls have been documented previously in the HEIA study [23]. One explanation could be that the intervention targeting SB (in 7^th^ grade only) was not extensive enough to influence the determinants of SB, since the components included only one computer tailoring session and one fact sheet to parents. No effect on perceived social inclusion at school was found either. However, all these determinants showed quite high baseline values (range 3.53-4.34, Table 3). Hence, a possible ceiling effect might also explain these post-intervention results.

In line with one other study among children [43], no effect modification by gender was found on the potential determinants. Results indicate that possible working mechanisms for PA change do not differ by gender. It also corresponds with findings for change in PA itself in children and adolescents. Van Sluijs et al. 2007 [18] concluded that most intervention studies observed no differential response by gender in PA change, and in a recent review by Cragg et al. 2011 [44] there was no consistent evidence of an association between gender and PA change among 10–13 year olds. Moderation effects of weights status on PA have been found in a previous cross-sectional study [22] and on mid-way effect on SB in a prospective study from the HEIA study [23]*.* However, no other studies have, to our knowledge, explored moderating effects of weight status and parental education on change in determinants for PA and SB among adolescents.

Our conflicting results on some of the determinants, especially among the overweight group, point to the importance of studying subgroup differences in the response to the intervention. Change in the expected direction in a determinant (the hypothesized mediator) is supposed to precede a desired change in behavior [3]. Hence, the no-effect and negative effect detected in determinants in some of the subgroups could work against desirable behavioral effects (i.e. in PA or SB).

### Perceived intervention dose received

The marked decrease in the proportion reporting a high intervention dose received from the mid-way (55.5%) to the post-intervention assessment (31.0%) could be one explanation for why effects were detected on several of the determinants mid-way, but not post**-**intervention. Furthermore, the adolescent reporting a high intervention dose received mid-way had significantly higher values on three out of four determinants than those reporting a low one. Post-intervention there were significant differences in favor of those with a high intervention dose received for enjoyment, social support from friends, perceived environmental opportunities for PA and perceived social inclusion at school (Table 6). This indicates that the intervention had an effect on change in these determinants among those most exposed to the intervention. However, the adolescents reporting a high intervention dose received mid-way showed significantly higher baseline values on self-efficacy and perceived social support from teachers compared to those receiving a low dose. Similar differences were found for enjoyment and perceived environmental opportunities for those adolescents receiving a high dose post-intervention (Table 6). Accordingly, for these determinants it seems as if the intervention increased the differences already present at baseline.

No differences in effects between the high and low intervention dose groups were seen for perceived social support from parents and perceived parental regulation of TV-viewing and computer/game-us (Table 6). Change in these parental related determinants was primarily targeted through the fact-sheets to the parents, and parental reported degree of exposure to the fact sheets would possibly be a better indication of the influence of implementation on these determinants.

However, overall the results from examining the intervention dose received suggest that the results revealing no effects in some of the outcomes in the main analyses might be due to an insufficient implementation of the intervention rather than insufficient intervention strategies. In support for this supposition, mid-way results from teacher reports of degree of implementation indicate that the overall degree of implementation was moderate [45]. It could be that the short e-mail reminders to the teachers to prompt the implementation of the various components were not sufficient to ensure a high degree of implementation over the course of the intervention.

There are both strengths and limitations to this study. The strengths include the high quality design and the theoretically based intervention in a large, long term study in a sample drawn from a region within a European country. Effects on potential determinants for both PA and SB were examined at two time points with high response rates. The analyses of moderating effects and corresponding subgroup differences added knowledge about intervention effectiveness across subgroups. As called for, the influence of perceived exposure to and participation in the intervention on the outcomes was explored. The limitations include the power analyses which were based on detecting change in PA and BMI, and not in the determinants [20]. However, the sample size of the study is larger than many previous studies including effect analyses on determinants [5,6,11]. The determinants assessed showed acceptable internal reliability at all time-points and test-retest reliability [22], but they might not have been sensitive enough for detecting change. The intervention was also extended to include an additional component (the computer tailoring program) in the last part. Therefore it is not possible to tease out whether the post-intervention results are related to this addition or to the intervention duration in itself. The wordings of the specific items measuring the determinants were directed towards PA and SB in general. It might have made it easier to detect intervention related changes in the determinants if they were phrased to match the intervention components more precisely since the different components were partly tailored to influence the behaviors in specific context. However, this was not possible in order to keep the questionnaire at reasonable length. While social desirability could have influenced the outcomes especially in the intervention group, the changes in the undesired direction for some of the outcomes go against such a line of reasoning*.* The seasonal difference between baseline (fall) and the two other data collection (spring) could also have affected the results. However, the weather conditions in Norway are quite similar for the two seasons in question and seasonal differences might be more pronounced in the actual behavior. The generalization of our findings might be somewhat weakened because a higher proportion than expected of the adolescents and parents declined to give consent. There might also be a possible attrition bias present due to the somewhat higher proportion of overweight adolescents and the lower values for perceived social inclusion found among those who only participated at baseline compared to several time-points. However, no differences between the control and intervention group were found among the non-responders at baseline (data not shown).

## Conclusion

The HEIA intervention did positively influence both psychological and social-environmental determinants, but also negative effects were observed. Further, effects on more of the determinants were seen mid-way than at the end of the intervention. In general, the effects obtained on the determinants were modest at best, and their practical relevance might be questioned. Still, from a public health perspective detecting even small favorable changes in factors with potential to influence PA may be important in order to inform intervention efforts at the population level.

Moderation effects and corresponding subgroup differences of both weight status and parental educational level were found. Most notably, the intervention did not seem to work equally well on change in enjoyment for the normal and overweight adolescents. More formative evaluation to better understand how to reach overweight adolescents seems needed. Finally, future research should continue to examine moderation effect of weight status on determinants of energy- balance related behaviors and examine how exposure to and participation in interventions influence intervention effectiveness.

## Competing interests

The authors declare that they have no competing interests.

## Authors’ contributions

All authors are responsible for the reported research. I.H.B worked on the statistical analyses, wrote the first draft of the manuscript and made the greatest contribution to the paper. N.L. was the project coordinator and participated in all parts of the work. K.I. K., L.F. A., S.A. and Y.O. were mainly involved in designing the study while M. B, M. G. and I.H.B. were mainly responsible for planning and conducting the data collections and the intervention. All authors provided critical revision of the paper, and read and approved the final manuscript.

## Supplementary Material

Additional file 1 **Table S1.** PA and SB intervention components and targeted determinants in the HEIA study. Click here for file
